# Transfer of heavy metals from soils to curly mustard (*Brassica juncea* (L.) Czern.) grown in an agricultural farm in Brunei Darussalam

**DOI:** 10.1016/j.heliyon.2021.e07945

**Published:** 2021-09-06

**Authors:** Adzrin Asikin Zunaidi, Lee Hoon Lim, Faizah Metali

**Affiliations:** a Chemical Sciences Programme, Faculty of Sciences, Universiti Brunei Darussalam, Jalan Tungku Link, Gadong BE1410, Brunei Darussalam; b Environmental and Life Sciences Programme, Faculty of Science, Universiti Brunei Darussalam, Jalan Tungku Link, Gadong BE1410, Brunei Darussalam

**Keywords:** Brassicaceae, DIM, EDEM, HRI, MTF, PCA

## Abstract

Determination of heavy metal concentrations in vegetables and agricultural soils is crucial because high levels of heavy metals could affect soil quality, crop production and safe consumption of crops. A field study was conducted to determine the heavy metal concentrations and their transfer from agricultural soils to different parts (leaf, stem, and root) of *Brassica juncea* (L.) Czern. In addition, potential health risks of contamination in the vegetables grown in the field were evaluated. Acid digestion method USEPA 3050B in combination with ICP-OES were used to analyze heavy metal (Al, Cd, Co, Cr, Cu, Fe, Mn, Ni, Pb and Zn) contents in both pre- and post-harvest soils and vegetable samples. Results showed that none of the heavy metals in soils had concentrations above the maximum safety limits based on the WHO, USEPA and CCME guidelines. Calculated metal transfer factor (MTF >1) showed *B. juncea* accumulated Cd, Co, Ni, Pb and Zn in leaves, stems and roots, but Cu and Mn, as well as Cr were only accumulated in stems and roots, respectively. There were variations in heavy metal contents between the different parts of *B. juncea*, but only Cd and Pb contents were above the maximum allowable limit recommended by FAO/WHO. PCA analysis was able to identify 4 major components corresponding to 38.38%, 28.98%, 14.39% and 10.67% of the total variance and PC1 was clearly associated to leaves of *B. juncea*. Based on the MTF values, only Cd was found to have a value of HRI >1 compared to the other heavy metals, implying potential health risk associated with long-term ingestion of the vegetable.

## 1 Introduction

Over the years, heavy metal contamination has potentially become an increasing global environmental and health concern, particularly in developing countries (Zhong et al., 2018; Rai et al., 2019; Yan et al., 2020). Heavy metals are metal elements that occur naturally in the environment comprising of essential heavy metals (eg. Cu, Fe, Mn, Ni and Zn) and non-essential heavy metals (eg. Al, Cd, Cr and Pb) (Santos et al., 2018). Essential heavy metals are normally required at optimal levels by plants and may play a role in plant growth and development, however, at elevated concentrations, they produce severe toxicity symptoms in plants (Arif et al., 2016; Rout and Sahoo, 2015). On the other hand, non-essential heavy metals have no biological and physiological functions in plants and are very toxic even at low concentrations (Singh et al., 2016a). In general, heavy metals in soils are derived from both natural and anthropogenic sources (Opaluwa et al., 2012; Alloway, 2013) where natural inputs are associated with minerals in the geological parent materials, while anthropogenic activities are linked to industrialization, urbanization and agricultural activities (Morgan, 2013; Zunaidi et al., 2020).

A significant source of heavy metal contamination in the human food chain originates from the bioaccumulation of heavy metals in food crops due to the fact that plants have the ability to absorb metals from soil, water and also air (Flores-Magdaleno et al., 2011; Chiroma et al., 2014). Heavy metals in soil migrate to plants through roots and leaves, with root uptake considered as the most predominant routes for metal uptake in plants (Chang et al., 2014; Garg et al., 2014). Accumulation of heavy metals in humans may occur via various pathways including the consumption of edible crops that are contaminated with heavy metals through food chain (i.e soil-plant-human transfer or soil-plant-animal-human transfer) (Wuana and Okieimen, 2011; Rehman et al., 2018), the inhalation of dust from polluted soil and the direct but accidental ingestion of metal-contaminated soil (Essien and Hanson, 2014).

Heavy metals are transported to plants in ionic forms, which can vary depending on the element (McLaughlin et al., 2011), while heavy metal speciation differs by food crop (Rai et al., 2019). According to McLaughlin et al. (2011), the intrinsic charge, valence and speciation of metal ion and soil properties, such as pH, redox potential, and clay and organic matter contents, could influence the behavior of metals and metalloids in polluted soils. The most important heavy metal element to consider in terms of food chain contamination are As, Cd, Hg, Pb and Se, which can disturb human metabolic activities, leading to diseases and even mortality (Rai et al., 2019). However, soil-to-plant metal transfer cannot be predicted solely by the contaminant's chemistry and behavior, as there are other significant processes regulating the aggregation of metals and metalloids in edible parts of vegetables, such as root uptake, root selectivity, ion interactions, rhizosphere processes, leaf uptake from the atmosphere, transpiration rate and plant partitioning (McLaughlin et al., 2011; Nedjimi, 2021). In addition, Biswas et al. (2018) reported that environmental related factors such as mineral contents of rainwater and groundwater may also influence the rate of metal uptake by plants.

Currently, there have been no studies on metal content and its transfer from soils to vegetables in Brunei, except for the study conducted by Zin et al. (2017), who reported heavy metals in paddy soils. It is therefore essential to gain insights into understanding heavy metal accumulation in vegetables and agricultural soils along with their possible health risks. Hence, the overall aims of this study are to investigate the heavy metal migration from soils to vegetables and assess the distribution of heavy metals in *Brassica juncea* (L.) Czern. grown on agricultural soils in Brunei Darussalam. The specific objectives are as follows: (1) to determine heavy metal concentrations in *B. juncea* (leaves, stems and roots) and agricultural soil, (2) to assess heavy metal migration and distribution from agricultural soils to different parts of *B. juncea*, (3) to evaluate the potential health risks of possible heavy metal contamination in the vegetables grown in the study area. This study will provide policymakers with baseline and reference data on the prevention and treatment of heavy metal contamination.

## 2 Materials and methods

### 2.1 Study area

**Figure 1:**
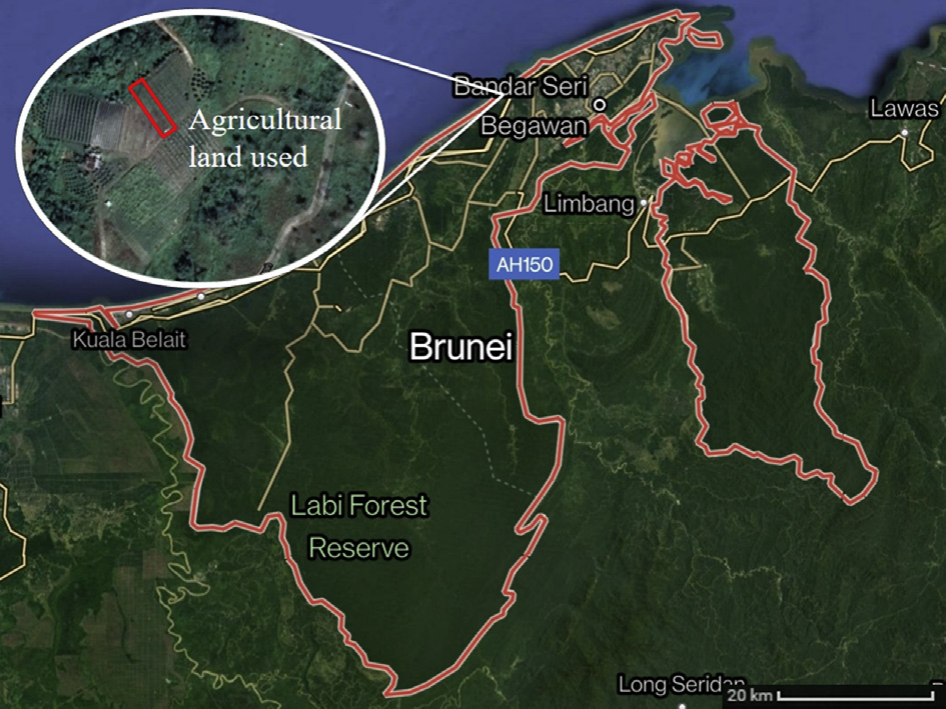
Map of Brunei Darussalam showing location of Bukit Agok agricultural farmland (Source: https://www.google.com/maps; Accessed: 22^nd^ June 2021).

The study area is located at Bukit Agok, which is one of the agricultural farms along Jalan Melati, off the Muara-Tutong highway in Brunei Darussalam (4°54′58.9″N, 114°47′44.4″E) (Figure 1), north-west of Borneo in South-East Asia. Bukit Agok (0.06 ha) is situated in the Brunei-Muara District, which has the most prevalent agricultural practices and activities in Brunei Darussalam (Agriculture and Agrifood Department, 2020). A variety of crops and vegetables are planted since 2015 and the local farmers usually sell them in the local markets in the Brunei-Muara District. For our study, *Brassica juncea* (L.) Czern. (curly mustard) from the family Brassicaceae was used as the study species.

**Figure 2:**
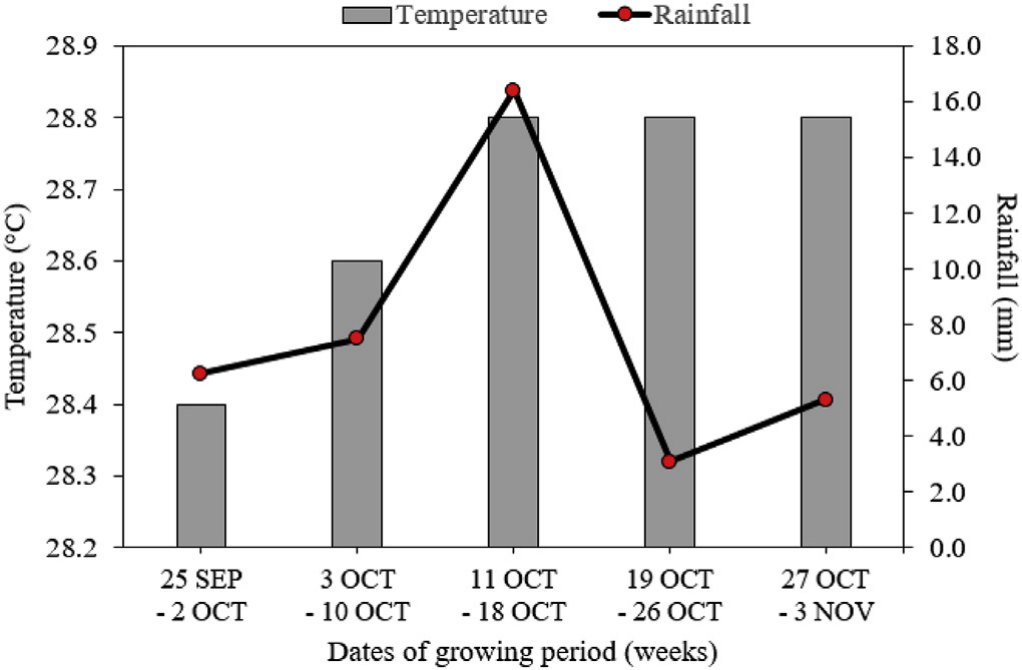
Average weekly temperature (°C) and rainfall (mm) during the growing periods of *Brassica juncea* (L.) Czern in September to November 2018. Data obtained from Brunei Darussalam Meteorological Department (BDMD), Ministry of Transport and Infocommunication, Brunei Darussalam.

Brunei has a humid and equatorial climate with high uniform average monthly temperature of 28.0 °C (min. 26.9 °C – max 28.7 °C) and average total rainfall of 3250 mm (min. 3134 mm – max. 3366 mm) in 2018 (Brunei Darussalam Meteorological Department, unpublished data). Average weekly temperature (°C) and rainfall (mm) during the growing periods of *B. juncea* (September to November 2018) is shown in Figure 2.

### 2.2 Study species, sampling and pre-treatment

The seeds of *Brassica juncea* (L.) Czern., which is an annual herbaceous plant were purchased from a local market in Brunei. *B. juncea* is a broad leafy vegetable which can be cooked or consumed raw (Mourato et al., 2015). Its seeds are important in mustard oil production and both plant and seeds were reported to have medicinal properties.

**Figure 3:**
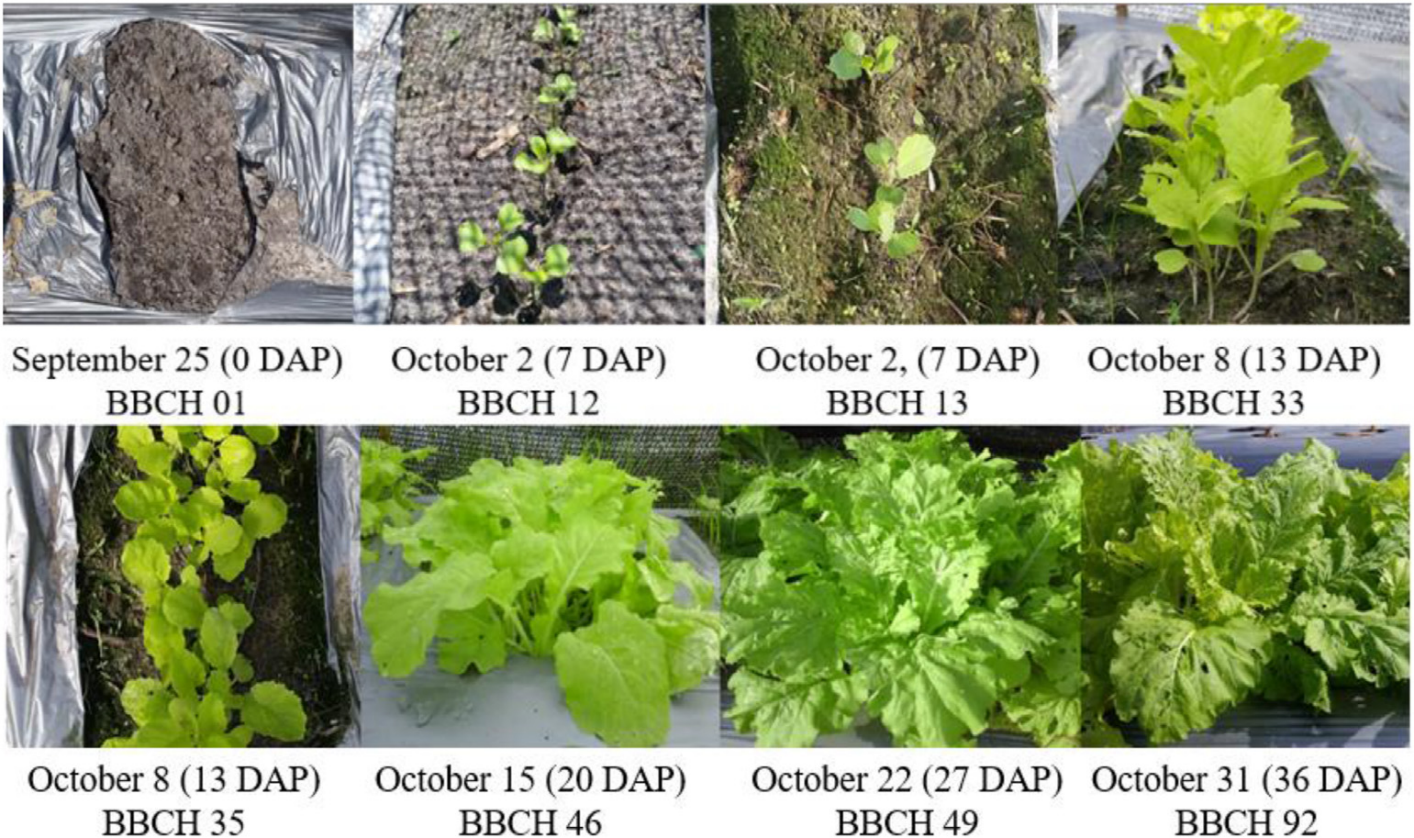
Phenological stages of *Brassica juncea* (L.) Czern. according to the BBCH scale (Feller et al., 2001): 01 (beginning of seed imbibition), 12 (second true leaf unfolded), 13 (third true leaf unfolded), 33 (leaf rosette has reached 30% of the expected diameter), 35 (leaf rosette has reached 50% of the expected diameter), 46 (60% of the leaf mass typical for the variety reached), 49 (typical leaf mass reached), 92 (leaves and shoots beginning to discolour). DAP – days after planting.

A total of 15 planting holes were set up on a rectangular raised agricultural bed (20 m × 0.6 m) in a 3 row x 5 column grid at Bukit Agok agricultural farmland (Figure 1). After three *B. juncea* seeds were sown in each planting hole, the planting bed, except the planting hole, was covered with a layer of black shade nets to avoid the vegetables from being directly exposed to sunlight that may affect their early growth. *B. juncea* took approximately 6 days to germinate and 5 weeks to mature (Figure 3). Inorganic phosphate (NPK) fertilizers 15.15.15 (4.16 g ± 0.62 g) and urea fertilizers (3.52 g ± 0.33 g) were added in the second week to ensure healthy growth of vegetables. A final harvest of all plants was conducted after 36 days in October 2018. All plants in each planting hole were bulked for chemical analysis, and for each vegetable part, the samples were analyzed in triplicates.

Pre-harvest (blank soil) and post-harvest soil samples were collected using a soil corer from each column grid before the vegetables were planted in September 2018 and after they were harvested in October 2018, respectively. The soil samples collected were stored in properly labelled plastic containers before taking them to the laboratory for further analysis. The purpose of analyzing pre- and post-harvest soil samples was to study the change in heavy metals after growing *B. juncea*. In addition, physicochemical properties of soils were also analyzed in triplicates (*n* = 15). The following properties are determined for the pre- and post-harvest soils: textural class (Storer, 2005), moisture content (Allen et al., 1989), pH (Hanlon, 2015), electrical conductivity (EC) (Van Reeuwijk, 2002), total organic carbon by loss-on-ignition method and organic matter content (Walkley and Black, 1934), soil nutrient content (Ca, Mg, Na, and K) and cation exchange capacity (CEC) (Gumbara et al., 2019), and the data are presented in Table 1.

**Table 1:** Selected physicochemical properties of soils (mean *±* standard deviation, SD) of pre-harvest and post-harvest soils from Bukit Agok agricultural farmland.

Soil parameters	Pre-harvest soil (*n* = 15)	Post-harvest soils (*n* = 15)
Taxonomy∗	Spodosols	
Textural class	Loamy sand	
Sand (%) (0.05–2.00 mm)	75.2 ± 0.0	75.9 ± 1.2
Silt (%) (0.002–0.050 mm)	22.1 ± 0.2	21.3 ± 1.2
Clay (%) (<0.002 mm)	2.7 ± 0.2	2.8 ± 0.0
% moisture	17.9 ± 0.7	19.7 ± 0.9
pH	5.2 ± 0.1	5.6 ± 0.1
Electrical conductivity (dS/m)	0.50 ± 0.22	0.30 ± 0.09
Total organic C (%)	4.2 ± 0.2	2.9 ± 0.2
Organic matter (%)	7.2 ± 0.4	5.1 ± 0.2
Ca (cmol/kg)	1.2 ± 0.3	1.0 ± 0.5
Mg (cmol/kg)	0.03 ± 0.03	0.03 ± 0.03
Na (cmol/kg)	0.71 ± 0.26	0.50 ± 0.28
K (cmol/kg)	0.05 ± 0.04	0.03 ± 0.02
CEC (cmol/kg)	1.98 ± 0.70	1.58 ± 0.80

∗ Soil taxonomy based on the United States Department of Agriculture (USDA) obtained from Grealish et al. (2007).

### 2.3 Analytical procedures

The extraction procedures were based on the United States Environmental Protection Agency (USEPA) method 3050B (USEPA, 1996). Prior to preparing the soil and vegetable samples, all glassware were soaked in 10% HNO_3_ for 24 h and rinsed with ultrapure water before proceeding with the analysis (Zin et al., 2017). Each of the soil and vegetable samples was oven-dried at 105 °C for 2 h to remove moisture and the dried sample was homogenized by grinding using a pestle and mortar and sieved. Prior to drying, the vegetable samples were separated into different parts (leaf, stem, and root) to study heavy metal accumulation from soils to *B. juncea*.

About 1 g of soil and vegetable samples were digested with 15 mL HNO_3_ (Sigma Aldrich, ACS reagent, 65%), 10 mL H_2_O_2_ (Sigma Aldrich, 30%), and 10 mL HCl (Sigma Aldrich, ACS reagent, 35.4%) for 2 h at 95 ± 5 °C. After cooling (*ca*. 24 °C), the samples were filtered and made up to volume using double distilled water. The samples were analyzed for Al, Cd, Co, Cr, Cu, Fe, Mn, Ni, Pb and Zn concentrations by inductively coupled plasma-optical emission spectroscopy (ICP-OES Thermo Scientific iCAP 6000 Series, USA). The accuracy of the instrument was checked by analyzing the samples in triplicates. The operating conditions of the ICP-OES used in this study is shown in Table 2.

**Table 2:** Operating conditions of ICP-OES.

Parameter	Elements
	Al (308.2), Cd (214.4), Co (228.6), Cr (283.5), Cu (224.7), Fe (371.9), Mn (403.0), Ni (221.6), Pb (220.3), Zn (481.0)
Output mode	Intensity
Plasma viewing mode	Axial
Spray chamber type	Glass cyclonic spray chamber
Instrument repeats	3
RF power	1150 W
Analysis pump rate	50 rpm
Auxiliary gas flow rate	0.5 L/min
Nebulizer gas flow rate	0.7 L/min
Flush pump rate	100 rpm
Pump stabilization time	15 s

### 2.4 Data analysis

#### 2.4.1 Metal transfer factor

The metal transfer factor (MTF) is one of the important parameters used in estimating the intake of metals by humans via consumption of vegetables (Garg et al., 2014). MTF or the amount of metal uptake by vegetables was evaluated using Eq. (1) below (Garg et al., 2014):

(1) $$Metal\;Transfer\;Factor\;\left(MTF\right)=\frac{C\;vegetables}{C\;soils}$$

where C_vegetables_ and C_soils_ are the concentration of metals in each leaf, stem, and root parts of vegetables (mg/kg) and the concentration of each relevant metal in the soils used to grow the vegetables (mg/kg), on dry weight basis, respectively.

#### 2.4.2 Daily intake of metals

The estimated daily oral intake of metals (DIM) from soils through vegetables was calculated using Eq. (2) described by Rehman et al. (2018):

(2) DailyIntakeofMetals(DIM)=DCV×MVC×Cf

where DCV, MCV and Cf are the average daily consumption of vegetables (0.345 kg/person/day for adults and 0.243 kg/person/day for children), mean metal concentration in vegetable (mg/kg) and conversion factor (0.085), respectively.

#### 2.4.3 Estimated daily exposure of metals

The estimated daily exposure of metals (EDEM) was to evaluate the intake of metals analyzed per body weight of an individual through planted vegetable consumption and Eq. (3) was used to calculate EDEM (mg/kg/day) (Rehman et al., 2018):

(3) $$Estimated\;Daily\;Exposure\;of\;Metals\;\left(EDEM\right)=\frac{\text{DIM}}{\text{Average ​body ​weight}}$$

where the average body weight of adults and children is 73 kg and 32.7 kg, respectively.

#### 2.4.4 Health risk index

The health risk index (HRI) in adults and children for this study was defined as the proportion of the estimated consumption of metals from vegetables or the estimated daily exposure of metals from vegetables (EDEM) against the standard reference oral dose or intake (RfDo) for each metal (USEPA, 2010; 2015). HRI can be calculated using Eq. (4) (Rehman et al., 2018):

(4) $$Health\;Risk\;Index\;of\;Metals\;\left(HRI\right)=\frac{\text{EDEM}}{\text{RfDo}}$$

where EDEM (mg/kg/day) and RfDo (mg/kg/day) are the estimated daily intake of metals and the reference oral dose or intake, respectively. According to USEPA (2010; 2015), the RfDo values are 0.0005, 1.5, 0.037, 0.7, 0.14, 0.02, 0.0035 and 0.3 mg/kg/day for Cd, Cr, Cu, Fe, Mn, Ni, Pb and Zn, respectively.

The USEPA has not derived the reference doses of the other heavy metals analyzed in this study, hence, only the selected heavy metals were determined for their HRI (USEPA, 2010; 2015). HRI determined the potential health risk of vegetables through consumption by both adults and children and an HRI value equals to or higher than 1 indicates that there is a potential health risk for consuming the planted vegetables by the selected heavy metals and that there should be a preventive measure taken to reduce the heavy metal contamination in vegetables and soils (Luo et al., 2011).

### 2.5 Statistical analysis

Statistical analyses were conducted in R 4.0.3 (Team, 2020). During model development, the heavy metal contents in pre- and post-harvest soils as well as in different parts of *B. juncea*, including leaf, stem, and root, were explored to confirm the normality of residuals and homogeneity of variances using Shapiro test and Levene's test, respectively and where necessary, data were log_10_-transformed. The significant differences in mean values of heavy metal elements between pre- and post-harvest soils and among different parts (leaf, stem, and root) of *B. juncea* were tested using parametric paired t-tests or non-parametric Wilcoxon signed-rank test and one-way analysis of variance (ANOVA) and TukeyHSD tests respectively, both at 5% significance level. Non-parametric Wilcoxon-signed rank test at 5% significance level was conducted as an equivalent test to ANOVA if the normality and homogeneity of variances of each dataset were violated. All heavy metal concentrations in *B. juncea* were used to derive a principal component analysis (PCA) of metal elements across the three different plant parts (leaf, stem, and root) (a matrix of 15 plant parts x 10 variables) in SPSS (SPSS, 2018).

## 3 Results and discussion

### 3.1 Heavy metals in soil and vegetable samples

Concentrations of heavy metals in the pre- and post-harvest soils used for planting *Brassica juncea* (L.) Czern. are presented in Table 3. Both the pre- and post-harvest soils contained the highest amount of Fe (215–323 mg/kg), followed by Al (204–215 mg/kg). However, most of the non-essential heavy metals, Cd, Cr, and Pb were found to be in the range of 1–3 mg/kg, while the essential heavy metals, Cu, Mn, Ni, and Zn were in the range of 1–76 mg/kg in both pre- and post-harvest soils. Beneficial heavy metal such as Co were found to be 1.5 mg/kg in both pre- and post-harvest soils. The concentrations of majority of elements decreased after the vegetable samples were harvested, except for soil Co, Cr, Fe, Mn, and Zn. However, none of the variations in soil metal concentrations were significantly different between the pre- and post-harvest soils. To assess the safety limit of the heavy metals detected in the soils, international agricultural soil quality guidelines recommended by the World Health Organization (WHO) (1996), USEPA (2007) and Canadian Council of Ministers of the Environment (CCME) (2014) are summarized in Table 4. Based on the standard quality guidelines, all 10 soil elements had mean values below the safety limits (WHO, 1996; USEPA, 2007; CCME, 2014). Interestingly, the Cd contents in both pre- (1.3 mg/kg) and post-harvest (1.1 mg/kg) soils were closer to the maximum permissible limit of 1.4 mg Cd/kg based on the standards set by CCME (2014), however, both values were below the USEPA guidelines of 3.0 mg Cd/kg (USEPA, 2007). The metal content in soils were probably attributed to the parent materials, topography, as well as content of iron oxides and hydroxides which may accumulate in the soil through adsorption (Shivanna and Nagendrappa, 2014). However, unfortunately, these properties were not investigated in this study.

**Table 3:** Heavy metal concentrations (mean ± standard deviation, SD) in pre-harvest and post-harvest soils grown with *Brassica juncea* (L.) Czern. The p-values were obtained after analyzing each heavy metal (row) using a paired t-test or a Wilcoxon-signed rank test at 5% significance level. Agricultural soil quality or permissible limit guidelines from the World Health Organization (WHO, 1996), United States Environmental Protection Agency (USEPA, 2007) and Canadian Council of Ministers of the Environment (CCME, 2014) were shown.

Heavy metals (mg/kg)	Soils		p-values	Agricultural soil quality guidelines (mg/kg)		
	Pre-harvest soils (*n* = 15)	Post-harvest soils (*n* = 15)		WHO (1996)	USEPA (2007)	CCME (2014)
Al	215 ± 43 (195–252)	204 ± 65 (161–241)	0.38	NA	NA	NA
Cd	1.3 ± 0.8 (0.8–1.9)	1.1 ± 0.9 (0.4–1.8)	0.22	NA	3.0	1.4
Co	1.5 ± 0.1 (1.3–1.5)	1.5 ± 0.5 (1.3–1.9)	0.29	NA	NA	40
Cr	2.7 ± 0.3 (2.4–2.8)	3.0 ± 0.9 (2.5–3.7)	0.11	100	150	64
Cu	11.2 ± 2.3 (9.2–12.5)	10.9 ± 7.8 (7.2–17.8)	0.62	30	140	63
Fe	251 ± 11 (244–257)	323 ± 81 (200–523)	0.22	NA	NA	NA
Mn	65.3 ± 6.1 (53.6–73.0)	75.8 ± 16.5 (38.8–125.2)	0.35	80	NA	NA
Ni	2.1 ± 0.3 (1.9–2.4)	1.9 ± 0.6 (1.4–2.1)	0.19	NA	75	45
Pb	1.9 ± 0.4 (1.5–2.2)	1.5 ± 0.6 (1.1–1.8)	0.17	NA	300	70
Zn	13.4 ± 3.9 (10.3–16.2)	18.9 ± 2.7 (7.7–31.6)	0.22	200	NA	250

NA – not available.

**Table 4:** Heavy metal concentrations (mean ± standard deviation, SD) in different parts (leaf, stem, and root) of *Brassica juncea* (L.) Czern. Different letters per heavy metal (row) represent significantly difference means after analyzing using parametric one-way ANOVA and TukeyHSD tests at 5% significance level. Agricultural vegetable quality or permissible limit guidelines from the Food and Agriculture Organization (FAO) and World Health Organization (WHO) (FAO/WHO, 1984) were shown below.

Heavy metals (mg/kg)	*Brassica juncea* (L.) Czern.			Agricultural vegetable quality guideline (dry weight) (FAO/WHO, 1984)
	Leaves (*n* = 15)	Stems (*n* = 15)	Roots (*n* = 15)	
Al	11.3 ± 0.2^ab^	8.2 ± 0.8^a^	47.2 ± 27.5^b^	NA
Cd	4.1 ± 1.7^a^	4.4 ± 0.9^a^	4.2 ± 0.4^a^	0.1
Co	3.2 ± 0.4^b^	3.1 ± 0.2^ab^	2.8 ± 0.4^a^	50.0
Cr	1.9 ± 1.7^a^	6.1 ± 0.0^bc^	31.6 ± 38.9^bc^	NA
Cu	11.3 ± 0.7^b^	4.5 ± 0.3^a^	5.3 ± 0.3^a^	73.0
Fe	70.6 ± 26.3^c^	26.2 ± 4.0^a^	40.9 ± 11.0^b^	425.0
Mn	145 ± 49^b^	28.3 ± 6.9^a^	37.0 ± 4.7^a^	500.0
Ni	3.1 ± 1.3^a^	3.0 ± 0.8^a^	3.5 ± 0.7^a^	67.0
Pb	4.8 ± 1.9^a^	4.8 ± 1.0^a^	4.4 ± 0.1^a^	0.3
Zn	35.9 ± 11.5^b^	30.6 ± 0.0^ab^	25.4 ± 7.1^a^	100.0

NA – not available.

The concentration of heavy metals in the different parts of *B. juncea* and agricultural vegetable quality guideline recommended by the Food and Agricultural Organization (FAO) and the WHO are shown in Table 4. There were variations in heavy metal contents between different parts of *B. juncea* with Al contents were much higher in roots. Co, Cu, Fe, Mn, and Zn contents were significantly greater in leaves than other parts (stem and/or root), however, for Al and Cr, the contents were significantly higher in stem and root parts. Cd, Ni and Pb contents were not significantly different among the plant parts.

Based on agricultural vegetable quality guideline recommended by FAO/WHO (1984), Co, Cu, Fe, Mn, Ni and Zn in all parts of *B. juncea* were below the permissible limits (Table 4). If the metal concentrations in plants are more than the maximum permissible limit, they could be deemed as phytotoxic and are harmful to humans if ingested via food chain (eg. Cu in Usman et al., 2019), and could negatively affect the physiological processes and metabolic activities of plants (eg. Zn in Kaur and Garg, 2021). Although the elements above are usually present in trace amounts, they can still contribute towards normal and healthy plant growth due to their functions as essential heavy metals in plants, which are required at a concentration of less than 100 mg/kg depending on the metal element (Marschner, 2012; Ahmed et al., 2020). For example, Cu is essential for enzymatic plant growth processes and a Cu concentration of less than 5 mg/kg in plants seemed to be insufficient for the growth of many vegetables as it could potentially affect the overall nutritional content and physiology (Printz et al., 2016; Sağlam et al., 2016).

The concentrations of Cd and Pb in all different parts of *B. juncea* were also found to contain above the maximum allowable limits for Cd (0.1 mg/kg) and Pb (0.3 mg/kg), respectively (Table 4). The Cd content in leaf, stem, and root of *B. juncea* (4.1–4.4 mg/kg) was at least 40 times the permissible limit of the standard guideline of FAO/WHO (1984). Similarly, Sterrett et al. (1996) reported Cd concentrations in lettuce ranging from 0.4 mg/kg to 3.5 mg/kg at different soil treatments were also above the permissible allowable limits recommended by FAO/WHO (1984). A study by Chaney et al. (1988); Chaney and Ryan (1994) documented that based on the toxic metal analysis in the bodies of local residents, an average concentration of 6 mg/kg Cd in lettuce did not pose potential health risk to farmers whose lifetime consumptions depended on the grown vegetables. Since the suggested average serving size of *B. juncea* is 56 g per person (Frey, 2020), this current finding could suggest that the high level of Cd in edible parts of *B. juncea* (4.1 mg/kg – 4.4 mg/kg) may not pose a major threat for human consumptions yet. However, one may need to limit the amount of intake of Cd-contaminated vegetable as high Cd consumption could affect human health (Rai et al., 2019; Huang et al., 2020). The concentrations of Cd in plants can be reduced by increasing soil pH, because alkaline soils could limit the Cd availability for uptake by plants (Scaccabarozzi et al., 2020).

The current study reported that the concentrations of Pb in all plant parts were in the range of 12–16 times more than the maximum allowable limits (Table 4). Several studies by Luo et al. (2011) and Garg et al. (2014) have also indicated the accumulation of Pb in leafy vegetables in their studies to be above the maximum allowable limits, with 4.4 mg/kg, 3.54 mg/kg and 2.2 mg/kg, respectively. The high Pb level in plants could be from anthropogenic activities such as addition of fertilizer to the soils, which could possibly result in a toxic accumulation of Pb in the soils (Zunaidi et al., 2020), posing potential health risk to humans if food chain is contaminated. It could also probably be due to physical contamination of vegetables by rainwater and dust towards the broad leafy vegetables (Luo et al., 2011).

**Figure 4:**
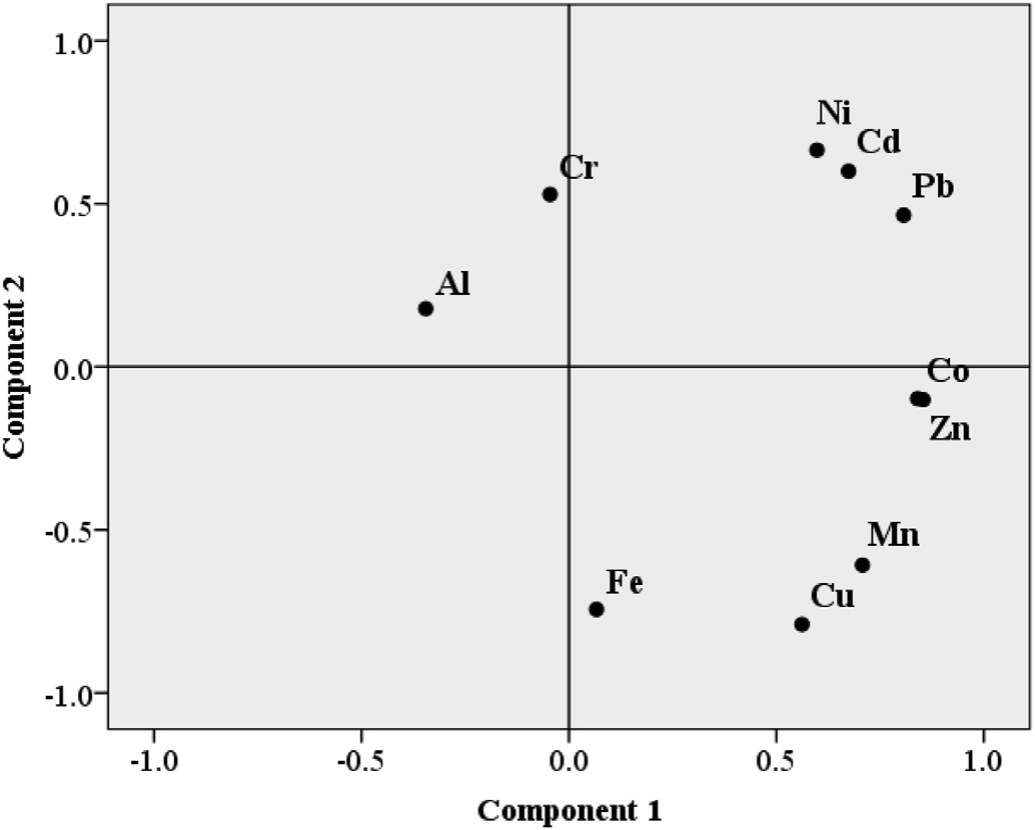
Biplot of scores for principal component analysis (PC) axes 1 (component 1) and 2 (component 2) from principal component analysis (PCA) of 10 metal elements across different parts (leaf, stem, and root) of *Brassica juncea* (L.) Czern. PC1 and PC2 accounted for the total variation of 38.38% and 28.98%, respectively.

**Figure 5:**
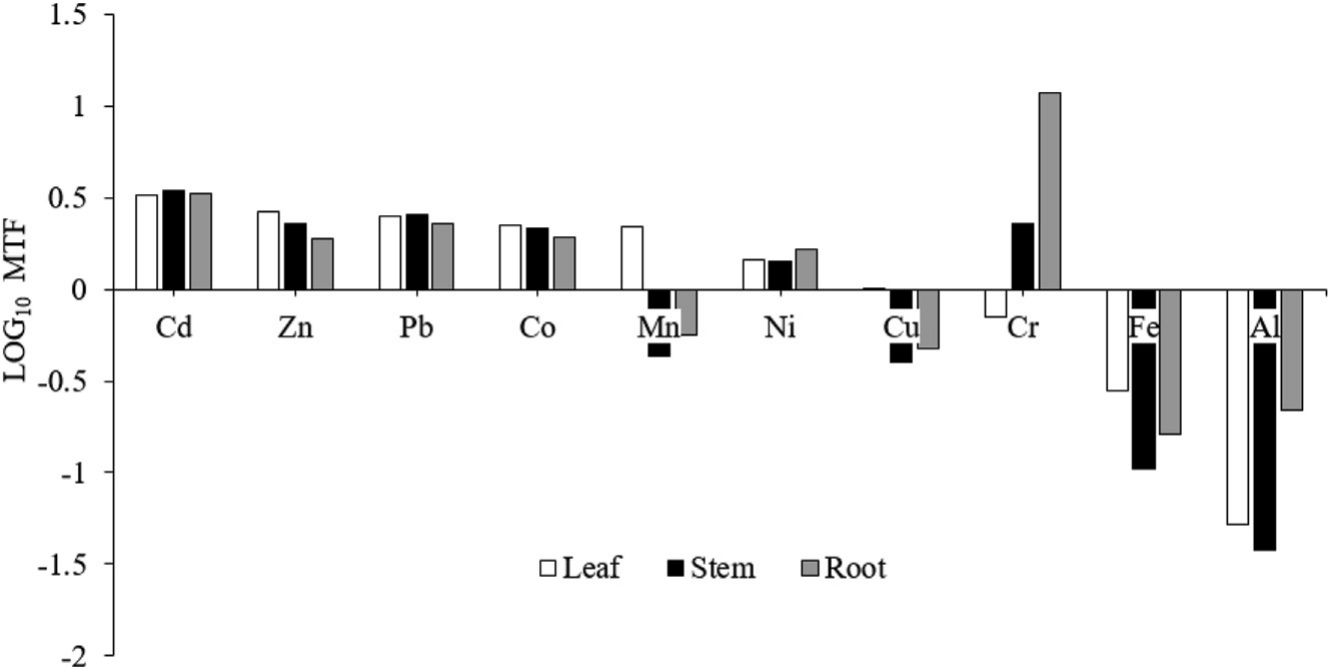
Metal transfer factor or MTF (log_10_) of metal elements from soils to different parts (leaf, stem, and root) of *Brassica juncea* (L.) Czern.

Principal component analysis (PCA) was performed and PC components with eigenvalues >1 and loading coefficients ≥0.40 are taken into consideration, following Dimitrijević et al. (2016). The PCA results are shown in Table 5 and Figure 4. The first four components (PC1 to PC4) explained 92.41% of the total variance, with, PC1, PC2, PC3, and PC4 accounted for 38.38%, 28.98%, 14.39%, and 10.67%, respectively. The PC biplot showed that the leaf parts were partitioned and differentiated based on Cd, Co, Cu, Fe, Mn, Ni, Pb and Zn concentrations. Majority of the heavy metals were clustered together on PC1, and were therefore associated with greater concentrations of Co, Cu, Fe, Mn, and Zn in leaves. Similar concentrations of Cd, Pb, and Ni were observed in all plant parts (Table 4). Most heavy metals were also observed to exhibit high and positive factor loadings in PC1, except Cu and Fe, and Ni, whose factor loadings were negatively associated and positively associated to PC2, respectively. PC3 and PC4 were positively associated with Al and Cr, respectively, and both Al and Cr were grouped together in the PC biplot (Figure 4) which was potentially related to high concentrations of Al and Cr in the root part of *B. juncea* (Table 4). This is consistent with the findings of Rout and Sahoo (2015), who revealed that crops could store Al in roots, while causing Al toxicity in roots. Since Cr is associated with greater concentrations in roots, there could be highly influence of Cr from soils (Figure 5).

**Table 5:** Eigenvalues from principal component analysis (PCA) of 10 metal elements across three different parts of *Brassica juncea* (L.) Czern. and percentage of total variance and cumulative variance explained by each principal component (PC) axis. Loadings and signs of the correlation coefficient of each metal element for the first four principal component axes were presented. Variables with the highest loadings are in bold.

Parameters	Principal component (PC) axes			
	1	2	3	4
Eigenvalue	3.84	2.9	1.44	1.07
Total variance explained (%)	38.38	28.98	14.39	10.67
Cumulative variance explained (%)	38.38	67.36	81.74	92.41
Metal elements	Component matrix			
Zn	**0.854**			
Co	**0.840**		-0.452	
Pb	**0.807**	0.465		
Mn	**0.707**	-0.608		
Cd	**0.674**	0.600		
Cu	0.562	**-0.790**		
Fe		**-0.744**	0.555	
Ni	0.598	**0.665**		
Al			**0.720**	
Cr		0.529		**0.753**

### 3.2 Dietary intake of heavy metals through vegetables

The migration pathways of metals from soils to vegetables could be revealed by computing MTF, which was based on the ratio of metal content in plant samples to the corresponding metals in the pre-harvest soils (Figure 5). MTF was considered to be one of the important parameters for evaluating exposure of heavy metals to humans via food ingestion and displays the availability of heavy metals in soils for uptake by plants (Garg et al., 2014).

If a plant has an MTF value of more than 1, it is considered as a hyperaccumulator (Oti, 2015). The sequence of MTF values in edible leaves and stems, and inedible roots varies (Figure 5). MTF values followed the order Cd > Zn > Pb > Co > Mn > Ni > Cu in leaves, Cd > Pb > Zn > Cr > Co > Ni in stems, and Cr > Cd > Pb > Co > Zn > Ni in roots, suggesting that *B. juncea* accumulated Cd, Zn, Pb, Co, and Ni in all plant parts. Strikingly, MTF of Cu and Mn (>1) was only noted for leaves, while MTF of Cr (>1) was observed for stems and roots, indicating that these elements are only accumulated in different plant parts. In many studies, it has been reported that *B. juncea* has MTF >1 for Cd, Cu, Ni, Pb, and Zn in both shoots and roots (Mourato et al., 2015; Sierra and Guerrero, 2021) as well as a promising plant for metal phytoremediation (Bassegio et al., 2020).

The difference in MTF values might be attributed to the physiology and metabolic activity of various plant parts, as well as climate conditions that could impact heavy metal transfer and mobility from agricultural soils to vegetables (Garg et al., 2014). Few studies have shown that higher concentrations of heavy metals in the agricultural soils would result in the greater probabilities of having high concentrations of heavy metals in the vegetables planted (Ramirez-Andreotta et al., 2013; Singh et al., 2016b).

It was also evident that adding fertilizers during planting could be a contributing factor to the hyperaccumulation of the metals in vegetables (Benson et al., 2014). The application of NPK and urea fertilizers to each plot of *B. juncea* could have contributed to the increased availability of metals in soils and metal accumulation in plants. In addition, there could be possible contribution from atmospheric deposition of trace metals, where heavy metals with MTF >1 such as Cd, Zn, Pb, Co, Ni, and Cu, in this study, were usually found to have the highest atmospheric deposition from traffic volume compared to other trace metals since the farmland is only about 1 km from the busy highway. Similar findings were also reported by Gunawardena et al. (2013) and Rehman et al. (2018) with MTF >1 for Pb, Cd, Ni, and Cu.

The migration pathways of metals to humans require exposure in terms of food-chain transfer of these heavy metals through oral ingestion of vegetables, dermal contact of contaminated soils, and dust inhalation (Garg et al., 2014). In this study, the RfDo, DIM, the EDEM and the HRI in both adults and children are summarized in Table 6.

**Table 6:** Reference dose of metals (RfDo), daily intake of metals (DIM), estimated daily exposure of metals (EDEM) and the health risk index (HRI) in adults and children.

	RfDo (mg/kg/day)	Adults			Children		
		DIM (mg/day)	EDEM (mg/kg/day)	HRI	DIM (mg/day)	EDEM (mg/kg/day)	HRI
Cd	0.0005	0.120	0.002	3.294	0.085	0.003	5.180
Cr	1.5	0.056	0.001	0.001	0.039	0.001	0.001
Cu	0.037	0.331	0.005	0.123	0.233	0.007	0.193
Fe	0.7	2.070	0.028	0.041	1.458	0.045	0.064
Mn	0.14	4.252	0.058	0.416	2.995	0.092	0.654
Ni	0.02	0.091	0.001	0.062	0.064	0.002	0.098
Pb	0.0035	0.141	0.002	0.551	0.099	0.003	0.866
Zn	0.3	1.053	0.014	0.048	0.742	0.023	0.076

RfDo value for each heavy metal was obtained in USEPA (2005).

In this study, EDEM values through ingestion of vegetables in adults and children were found to be below 1, with Cd having the highest estimated exposure of 0.002 mg/kg/day and 0.003 mg/kg/day in adults and children, respectively (Table 6). The estimated exposures between adults and children in this study was found to be consistent with the findings from Tewari and Pande (2013). This study found that Cd has the highest DIM, EDEM, and HRI for *B. juncea* with HRI of 3.294 and 5.180 in adults and children, respectively. The overall sequence for the EDEM and HRI in both adults and children through ingestion of the cultivated vegetables were in the order of Cd > Pb > Mn > Cu > Ni > Zn > Fe > Cr.

The HRI values of >1 for Cd in vegetable samples indicated that there is a possible health risk in consuming the analyzed vegetable samples continuously over the years (Muhammad et al., 2011) because of the persistence and long residence times of Cd and other heavy metals in soils (Selvi et al., 2019). The high HRI in all of the heavy metals analyzed in children than the adults suggested that children were more easily exposed to these toxic heavy metals compared to adults through consumption of these vegetables (Wang et al., 2021). Therefore, a proper assessment of heavy metals on vegetable consumption needs to be regulated as a safety risk measurement especially to the vulnerable group in order to protect the population from these exposures (Tariq, 2021).

## 4 Conclusion

The agricultural soils in this study did not contain heavy metals, whose concentrations are above the maximum safety limit according to the CCME, USEPA, and WHO guidelines. Based on the criterion of MTF >1, *Brassica juncea* (L.) Czern. accumulated Cd, Co, Ni, Pb and Zn in leaves, stems, and roots but Cu and Mn, as well as Cr were only accumulated in leaves, and stems/roots, respectively. Only Cd and Pb contents of *B. juncea* were above the maximum allowable limit recommended by FAO/WHO. PCA results showed Cd, Co, Mn, Pb and Zn exhibiting high component scores in PC1, which was associated to leaves. The EDEM results showed that both adults and children were not exposed to the heavy metals through consumption of *B. juncea*, but the HRI for Cd in both adults and children were found to be >1, suggesting that there is a potential risk for long-term consumption of the contaminated vegetable. Therefore, it can be concluded that monitoring of heavy metals in agricultural soils and food crops should be conducted regularly to assess the uptake of heavy metals and public health risks posed to consumers. There is also a need to provide proper assurance to the public that they are protected from consuming contaminated food that might cause undesirable health implications.

## Declarations

### Author contribution statement

Adzrin Asikin Zunaidi: Conceived and designed the experiments; Performed the experiments; Analyzed and interpreted the data; Contributed reagents, materials, analysis tools or data; Wrote the paper.

Lim Lee Hoon; Faizah Metali: Conceived and designed the experiments; Analyzed and interpreted the data; Contributed reagents, materials, analysis tools or data; Wrote the paper.

### Funding statement

This study was supported by the Faculty of Science Block Fund (UBD/RSCH/1.4/FICBF(b)/2018/006), 10.13039/100009100Universiti Brunei Darussalam, Brunei Darussalam. A. A. Zunaidi study was funded by the Ministry of Education, Brunei Darussalam.

### Data availability statement

Data will be made available on request.

### Declaration of interests statement

The authors declare no conflict of interest.

### Additional information

No additional information is available for this paper.
